# Floor Feeding Sows Their Daily Allocation over Multiple Drops per Day Does Not Result in More Equitable Feeding Opportunities in Later Drops

**DOI:** 10.3390/ani8060086

**Published:** 2018-06-05

**Authors:** Megan Verdon, Natalia Zegarra, Rutu Achayra, Paul H. Hemsworth

**Affiliations:** 1 Animal Welfare Science Centre, Faculty of Veterinary and Agricultural Sciences, University of Melbourne, Victoria 3010, Australia; natelianazeg@gmail.com (N.Z.); rutu.acharya@unimelb.edu.au (R.A.); phh@unimelb.edu.au (P.H.H.); 2 Tasmanian Institute of Agriculture, College of Sciences and Engineering, University of Tasmania, Tasmania 7320, Australia

**Keywords:** aggression, feeding behavior, floor feeding, sow

## Abstract

**Simple Summary:**

Floor feeding is one of the cheapest and simplest methods of feed delivery for groups of commercial gestating sows, but results in high levels of competition for feed. Consequently, feed intake and weight gain are reduced for low-ranking sows in floor-feeding systems. More equitable feeding opportunities may be achieved by providing floor fed sows their daily allocation over multiple feed drops per day. This study recorded (over two gestations) the aggressive and feeding behavior of sows that were floor fed four times a day. High-ranking sows spent the most time feeding where the majority of feed was distributed. All other sows fed opportunistically, consuming what they could from between and around high-ranking females. The lowest ranking sows spent more time than middle and highly ranked sows avoiding the feeding area. These relationships were true regardless of day, feed drop, or gestation. Further research is necessary to ensure that all sows are able to feed with less risk to their welfare, or, alternatively, determine whether variation in feed intake is a feature of floor feeding systems per se. In terms of accessing the feeding area, this research has broad implications for most feeding systems.

**Abstract:**

This research studied whether floor feeding group-housed sows their daily allocation over multiple feed drops per day provides more equitable feeding opportunities in later drops. Over four time replicates, 275 sows were mixed into groups of 10 for both their first and second gestations (200 sows/gestation, 126 sows observed in both gestations). The feeding behavior of individual sows was recorded for 10 min following each of four feed drops per day (0730, 0900, 1100, 1500 h) on days 2, 9 and 51 post-mixing. The location of feeding sows (i.e., feeding in areas associated with high, reduced or little/no food availability) was also recorded. Sow aggressive behavior on day 2 was used to classify sows as dominant (D), subdominant (SD), or submissive (SM). Dominant sows spent the most time feeding in areas of high-food availability (gestation 1, *p* < 0.001; gestation 2, *p* = 0.023); SD sows fed more frequently than D sows from areas of reduced food availability (gestation 1, *p* = 0.001; gestation 2, *p* = 0.025); and SM sows performed more feeding behavior in areas of little/no food availability (gestation 1, *p* < 0.001; gestation 2, *p* < 0.001). These relationships did not change over feed drops or days in either gestation (*p* > 0.05). Further research on the management and design of floor feeding systems is required, with a particular emphasis on increasing accessibility to sows that avoid the feeding area.

## 1. Introduction

Sow aggression continues to challenge the success of group-housing sows post-insemination. While aggression observed post-mixing—associated with the formation of dominance relationships—is particularly intense, the establishment of a dominance hierarchy does not eliminate aggression between group-housed sows. Sows housed in stable groups will continue to show aggression over competition for access to restricted resources [1]. Commercial gestating sows are commonly fed a restricted diet that is unlikely to result in satiation, and consequently the restricted resource sows most frequently compete over is food [2,3].

The type of feeding system utilised affects competition for feed, and consequently the level of sow aggression associated with feeding [4]. Dropping feed directly into the pen floor (i.e., floor feeding) fulfils some elements of natural feeding behavior by allowing sows to feed simultaneously [5]. However, the resulting competition for access to, or defense of, food results in aggression immediately following feed delivery [1,3], which continues until the majority of feed has been consumed [6]. Consequently, when fed from the floor, submissive sows may sacrifice the opportunity to feed in an attempt to avoid receiving aggression [1,7]. This often results in low-ranking sows having reduced intake and gaining less weight throughout gestation than higher-ranking pen mates [1,8]. 

Feeding sows individually (e.g., using an electronic feeding station (ESF) or feeding stalls) has been advocated by researchers on the premise that it reduces aggression associated with feeding and allows for better control of individual sow intake (see Bench et al. [9]). However, the low costs associated with the implementation and management of floor feeding systems continue to make them an attractive option for commercial pig farmers. Direct comparisons of sow aggression in floor feeding and ESF systems are lacking (reviewed by Verdon et al. [5]). Increased vulva bites and injuries are reported when sows are fed in full body feeding stalls without back gates [10] and ESF stations [11], compared to floor or trough feeding systems. This suggests that gaining access to individual feeding systems can also lead to competition and aggression [9].

Although feeding sows once a day is common [6,12,13], floor feeding sows their daily allocation over multiple feed drops per day and may create more opportunities for subordinate sows to feed in later drops. A preliminary study conducted by Rault et al. [14] suggested that, when fed over four feeding drop per day, dominant sows are present less frequently in the third than the first drop. Verdon et al. [15] reported that the intensity of aggression received by subordinate sows reduces with subsequent feed drops. Further, Schneider et al. [16] found that increasing feeding frequency from two to six drops reduced vulva and skin lesions as well as the frequency of culling for lameness and leg injuries in sows, but not gilts.

This study examined the relationships between aggressive and feeding behavior in group-housed sows when floor fed over multiple drops per day. The hypothesis tested was that feeding sows their daily ration over several smaller meals throughout the day will provide more equitable feeding opportunities, as indicated by a reduction in time spent by dominant sows, an increase in time spent by subordinate sows, feeding in successive feed drops both within days, and between days 2, 9 and 51 of gestation.

## 2. Materials and Methods

### 2.1. Ethical Statement

All animal procedures were conducted with prior institutional ethical approval under the requirement of the New South Wales Prevention of Cruelty to Animals Act (1979) in accordance with the National Health and Medical Research Council/Commonwealth Scientific and Industrial Research Organization/Australian Animal Commission Australian Code of Practice for the Care and Use of Animals for Scientific Purposes (NHMRC, 2013). 

### 2.2. Facilities

This study was conducted over 16 months in a gestation unit of a large commercial piggery in southern New South Wales, Australia. The naturally ventilated 6-m long and 19-m wide building was equipped with adjustable blinds. Overhead water sprinklers covered 50% of the slatted floor area of the pens and were activated (3 min on and 15 min off) when the internal temperature exceeded 26 °C. The maximum and minimum mean daily ambient temperatures for spring, summer, autumn, and winter of the experimental period were 21.3 and 8.5 °C, 29.2 and 15.2 °C, 21.3 and 7.9 °C, and 15.6 and 3.9 °C, respectively. Within the unit, 12 pens (3.7 by 4.8 m) were used. Each pen had partially slatted floors (50%) with a solid cement lying/feeding area and a slatted dunging area and was fitted with two overhead feed droppers and one nipple drinker. One video camera with built-in infrared lights was positioned above each pen and recorded from 0700 to 1700 h on the second day of mixing (labelled day 2) and days 9 and 51 after mixing. The camera covered most of the pen floor area (14 m^2^, 81% of the pen); however, some areas in the corners of the pens could not be observed. Importantly, the area of the pen floor where feed was delivered was within the field of view of the camera and this is where most of the sow interactions at feeding occurred.

### 2.3. Animals and Experimental Design

This opportunistic study utilised video data collected in a previous research project [1] using overhead video cameras (720p AHD outdoor camera with IR, 3.6 mm, QC-8637; Jaycar electronics, Australia) that were connected to a digital video recorder (TECHview 16 Channel stand-alone DVR, model QV-3039; Techbrands). A total of 275 pregnant Large White × Landrace sows (*Sus scrofa*) were used in this study so that 200 gilts (50 gilts per replicate) in 4 time replicates were studied in their first gestation and 200 sows in 4 time replicates were studied in their second gestation (200 animals per gestation with 126 animals common to both gestations). Gilts detected in oestrus from 32 weeks of age were transferred from groups of 30 gilts to stalls for insemination. Gilts were twice artificially inseminated (morning/afternoon insemination routine) and, within 7 d of insemination, were randomly mixed into groups of 10 (space allowance of 1.8 m^2^/gilt) between 0800 and 1300 h. Before mixing, symbols were sprayed on the backs of gilts allowing for individual identification. One week before farrowing, gilts were moved to farrowing stalls where they remained until piglets were weaned at 25 d of age. After piglets were weaned, the parity 1 sows were housed in mating stalls, again twice artificially inseminated (morning/afternoon insemination routine), and within 7 d, were randomly mixed into groups of 10 (space allowance of 1.8 m^2^/sow). Females were allocated to different groups for their second gestation and remained in these groups for the remainder of the gestation. On average, the maximum number of sows in a group that had been housed together in the first gestation was 2.4 (range 0–4). The same farrowing management as for the first gestation was applied. For convenience, in the remainder of this paper, gestating gilts will be referred to as sows. The gestation number of the sow will reflect her parity status: nulliparous or primiparous. During gestation, sows were fed a standard commercial gestation pelleted diet (13.1 MJ/kg DM and 12.8% protein; 31.3 kg per feeder per drop at 2.5 kg per sow per d). Feed was delivered to the floor in four feed drops (at approximately 0730, 0930, 1100, and 1500 h). One nipple drinker that was located on the wall over the slatted flooring of each pen provided water ad libitum.

### 2.4. Measures Recorded

#### 2.4.1. Feeding Behavior

From video records, the feeding behavior of individual sows was observed using instantaneous point sampling [17] with 30 s intervals over four, 5 min time blocks (i.e., a total of 20 min). Recording commenced 30 s after the delivery of each of the four feed drops per day, at the day after mixing (day 2), day 9 and day 51 (40 possible sample points per sow, per feed drop, per day). Video quality and high animal activity made it difficult to determine if a sow was actually feeding (i.e., picking food up, chewing). Thus, a sow was recorded as performing feeding behavior if her head was down and she was rooting the ground/feed, or if she was stationary in the area that the majority of feed was delivered with her head up. The latter was included following preliminary observations that identified sows occupying a position over the area of feed delivery taking mouthfuls of pellets and lifting their heads to masticate. If a sow was performing feeding behavior, her location in the pen was recorded as either (1) directly under the feed hopper (i.e., high feed availability, HF; a reverse cone shape visually estimated to be approximately 1 m diameter, occupying an approximate 0.79 m^2^ floor space, per feeder), (2) on the cement flooring but excluding the area directly under the feed hopper (i.e., reduced feed availability, RF), or (3) on the slats at the back at the pen (i.e., scarce or no feed availability, NF). The location of sows that were not performing feeding behavior was not recorded.

#### 2.4.2. Aggressive Behavior at Feeding

Aggressive behavior of individual sows was observed continuously for 30 min after each of the four daily feed drops on days 2, 9 and 51. Aggressive behavior was defined as bites, presses, and knocks [18] and also included fights, which were defined as aggressive interactions involving the same pair of animals and that continued for at least a 5 s duration [1]. During fights, a bout criterion interval of 5 s was chosen to separate one bout of aggressive behavior from another bout [19]. The numbers of aggressive acts delivered and received by each individual sow during the observation period were recorded. Only when the full head of the attacking animal and the identifying symbol of the animals delivering and receiving aggression were clearly in the field of view were aggressive interactions recorded. From the observations of aggression at feeding on day 2, sows were classified as “dominant” if they delivered more aggression than they received on day 2, “subdominant” if they received more aggression than they delivered on day 2, and “submissive” if they delivered very little or no aggression relative to aggression received on day 2 (that is, the ratio of aggression delivered to aggression delivered + aggression received ≤ 0.05). Aggressive behavior on day 2 was used because aggression between group-housed sows that are restrictively fed is most pronounced early after grouping [20]. This aggression classification is the same as that used by Verdon et al. [1], and similar to that devised by Mendl et al. [21] and used later by Zanella et al. [22], but these researchers used displacements rather than aggression. Nonetheless, each study found that most animals were classified as subdominant (44.9–64.9%) with fewer numbers of submissive (18.9–20.8%) and dominant (16.2–34.3%) sows. Verdon et al. [1] reported a low level of consistency in sow aggression classification between gestations 1 and 2, although individual sow aggressive behavior is relatively consistent within each gestation [15]. Changes in the aggressive behavior of sows over the four feed drops per day are reported elsewhere [15]. 

### 2.5. Statistical Analysis

Due to removal of animals for reproductive failure or poor health (injury, illness, death), and to ensure each gestation had 200 animals at mixing, a total of 275 animals were selected for the study. Some animals were observed in the first gestation and not the second, and vice versa, but there were 126 animals common to both gestations.

The number of sows and pens observed at each day of each gestation are reported in Table 1. In the first gestation, a technical malfunction meant there was no day 51 behavioral data for replicate 3, while an operational error meant that the fourth feed drop was not delivered on day 2 of replicate 2 or day 51 of replicate 1. In the second gestation, the same operational error meant that the fourth feed drop was not delivered on days 2 or 51 for replicate 3. On day 51 of gestation 2, one pen in replicate 3 broke a gate and mixed with non-experimental pigs, so feed drops 3 and 4 were excluded from analysis for this pen. One gestation 2 sow escaped from the pen before any data could be obtained.

**Figure 1:**
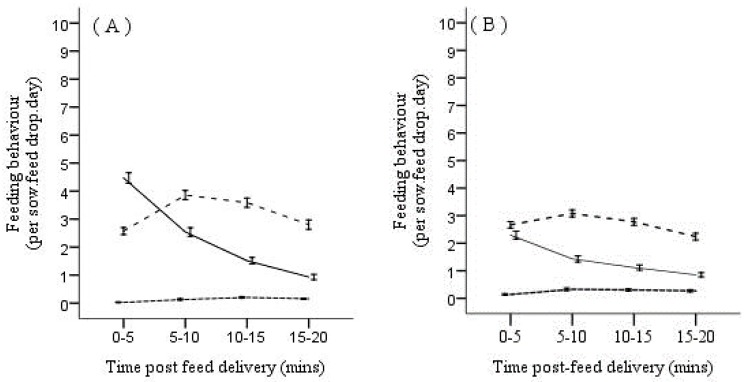
The average (±95% confidence interval) number of times sows were recorded performing feeding behavior (per sow, feed drop and day) in areas of the pen associated with high (▬), reduced (- - -) and no (···) feed availability recorded over four, 5-min time periods immediately following feed delivery in gestations 1 (**A**) and 2 (**B**).

The statistical package used for all analyses was SPSS 23.0 (SPSS Inc., Chicago, IL, USA) and the unit of analysis was always the individual gilt/sow. A preliminary inspection of the data revealed that the time sows spent feeding in the area of high feed availability declined, and the time spent feeding in the area of reduced feed availability increased, between 5 min to 10 min post-feed delivery (Figure 1). This suggests that the majority of feed was consumed within 10 min of delivery, and so only the first 10 min of feeding behavior data was used in statistical analysis.

Data on the feeding behavior of sows per feed drop within days represented a count of occurrences (i.e., number of observations that a sow was feeding) in a fixed period of time (i.e., total of 10 min post-feed delivery). As such, GLMMs with an underlying Poisson distribution and log link function were developed to relate the total number of times SM, SD and D sows were observed displaying feeding behavior in each of the four feed drops on days 2, 9 and 51. Separate models were developed for locations associated with high (HF), reduced (RF) or no (NF) food availability and for gestations 1 and 2. For all analyses, feed drop within a day (1, 2, 3, 4), day post-mixing (2, 9 and 51), and aggression classification (SM, SD, D), as well as their 2- and 3-way interactions, were included in the model as fixed factors. Repeated observations of the same sow over (1) feed drops within days, and (2) days within gestations, were accounted for with a first order auto-regressive correlation structure. Group/pen (nested within replicate) and replicate were included in the model as random blocking factors. When an interaction was not significant, it was removed from the model so that significant effects could be more reliably interpreted. Where there were significant main or interactive effects (*p* < 0.05), the LSD test determined where estimated marginal means differed.

## 3. Results

**Figure 2:**
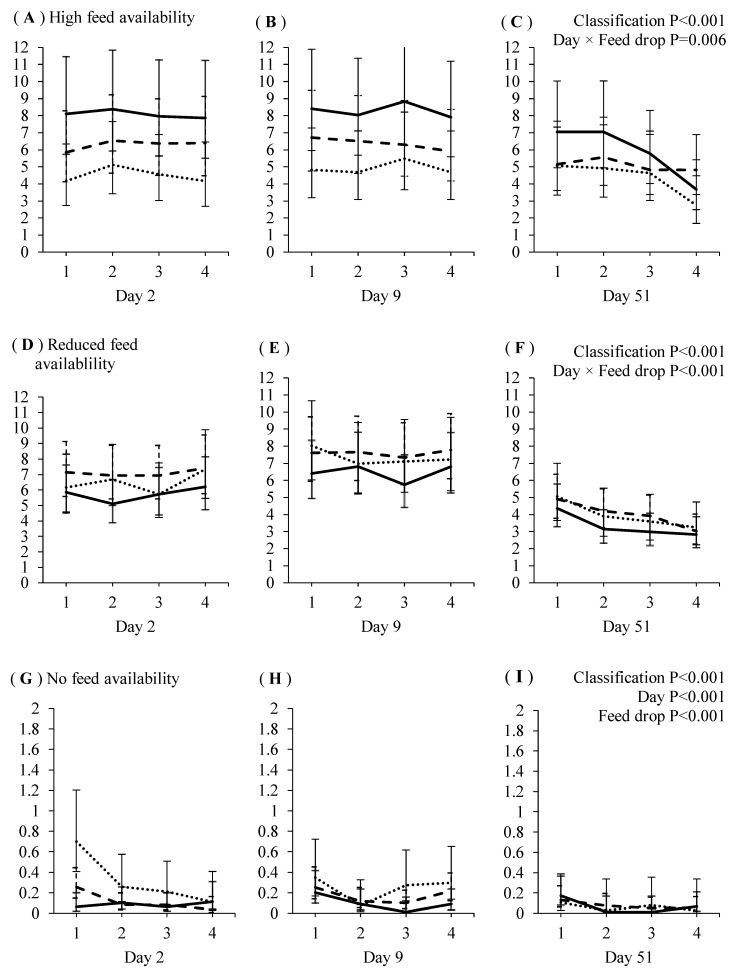
Gestation 1: The average (±95% confidence interval) number of times dominant (▬), subdominant (---) and submissive (···) sows were recorded showing feeding behavior (y-axis) in areas associated with high (**A**–**C**), reduced (**D**–**F**) or no (**G**–**I**) food availability, when fed over four feed drops (x-axis) on days 2, 9 and 51 post-mixing.

**Figure 3:**
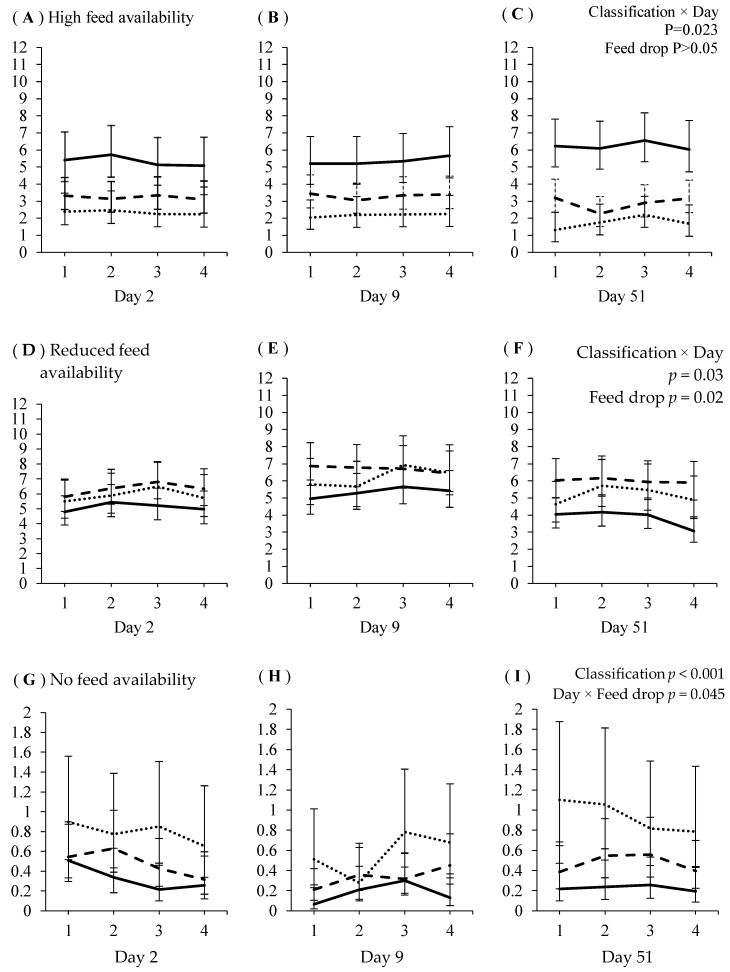
Gestation 2: The average number (±95% confidence interval) of times dominant (▬), subdominant (---) and submissive (···) sows were recorded showing feeding behavior (y-axis) in areas associated with high (**A**–**C**), reduced (**D**–**F**) or no (**G**–**I**) food availability, when fed over four feed drops (x-axis) on days 2, 9 and 51 post-mixing.

Relationships between sow aggression classification and feeding behavior over four feed drops delivered on days 2, 9 and 51 of gestations 1 and 2 are presented in Figure 2 and Figure 3. There were no main effects of feed drop or interactive effects of aggressive classification × feed drop on the time sows spent feeding directly under the feed hoppers (i.e., HF), or at locations associated with reduced feed availability (i.e., RF), in either gestation 1 or 2 (*p* > 0.05).

In both gestations, dominant sows spent the most, and submissive sows the least, time feeding at the HF location (gestation 1: aggression classification F_2,2259_ = 24.6, *p* < 0.001; gestation 2: aggression classification × day F_4,2199_ = 2.9, *p* = 0.023). The aggression classification × day interactive effect in gestation 2 is due to dominant sows spending more time, and submissive sows less time, feeding at the HF location on day 51 compared to days 2 and 9. The time sows spent feeding at the HF location in gestation 1 was not affected by feed drop on days 2 and 9, but declined with subsequent feed drops on day 51 (feed drop × day F_6,2259_ = 3.0, *p* = 0.006).

Subdominant sows spent more time feeding at the RF location than dominant, but not submissive, sows in gestation 1, although submissive and dominant sows did not differ (aggression classification F_2,2259_ = 6.63, *p* = 0.001). There was a significant aggression classification × day interaction effect on time feeding at the RF location in gestation 2 (aggression classification × day F_2,2199_ = 2.8, *p* = 0.025). Regardless of day, subdominant sows spent more time feeding at RF than dominant sows. The time submissive sows spent feeding at RF did not differ from dominant or subdominant sows on day 2 of gestation 2. On day 9, submissive sows spent more time feeding at RF than dominant (but not subdominant) sows. On day 51, submissive sows fed at RF less frequently than subdominant sows, but more frequently than dominant sows. The time sows spent feeding at the RF location in gestation 1 increased from feed drop 3 to 4 on day 2, was not affected by feed drop on day 9, and declined over subsequent drops on day 51 (day × feed drop F_6,2259_ = 5.4 *p* < 0.001).

In gestations 1 and 2, submissive sows spent the most time, and dominant sows spent the least, feeding on the slats (NF) at the back of the pen (gestation 1 aggression classification: F_2,2265_ = 9.3 *p* < 0.001; gestation 2: aggression classification F_2,2197_ = 27.1 *p* < 0.001). In gestation 1, time feeding at NF was higher during feed drop 1 than drops 2, 3 and 4 (feed drop number F_3,1116_ = 15.1 *p* < 0.001) and was higher on days 2 and 9 than day 51 (F_2,1346_ = 5.1 *p* = 0.006). In gestation 2, time spent feeding at the NF location declined over the four feed drops on day 2, but on day 9, time feeding at NF was higher in drops 3 and 4 than in drop 1, whereas on day 51, time feeding at NF was not affected by drop (feed drop × day F_6,1689_ = 2.8 *p* = 0.01).

## 4. Discussion

To the best of our knowledge, this is the first published research in over two decades to examine variation in the feeding behavior of floor-fed gestating sows. For the purpose of this study, a sow was defined as feeding if her head was down and she was rooting the ground/feed, or if she was stationary in the area that the majority of feed was delivered with her head up. Aside from Csermely and Wood-Gush [23], no other study has examined the relationship between individual sow aggression and feeding behavior when the daily feed allowance is delivered over multiple feed drops per day.

The results of this research indicate that dominant sows spend the most time feeding directly under the feed hoppers, and the least time feeding in areas of reduced or no feed availability. Simple drop feeding systems distribute feed onto the floor in a reverse cone shape, with the highest volumes of feed directly under the feeder. Thus, sows that spend significantly more time feeding directly under the feeder are likely to have the highest intake of food per unit of time [23]. Subdominant sows fed more frequently than dominant sows from areas of presumably reduced feed availability (i.e., around the edges of the area where the majority of feed was delivered). Submissive sows fed on the slatted area of the pen more often than subdominant and dominant sows. This suggests that submissive sows avoided the feeding area despite being motivated to feed, as it is unlikely that food was present in this slatted area of the pen. Similar results were reported by Csermely and Wood-Gush [23] and Brouns and Edwards [6] in studies with less replication than the present one (4 and 2 pens, respectively).

The relationships between aggression classification and feeding behavior in the present study did not change over feed drops or days post-mixing. These results are comparable to studies that floor fed sows once [6] and twice [23] per day. Anecdotal reports suggest that dominant sows spend more time delivering aggression in defense of food than consuming it [3,6,23]. Other research have found sow aggression at the group level to decline [7], and aggression delivered by dominant sows, and received by dominant, subdominant and submissive sows, to decline over multiple feed drops within a day [15], while sows sustain fewer skin injuries when fed six times rather than twice per day [16]. It has previously been hypothesised that dominant sows become satiated after the first feed drop of the day, providing subdominant and submissive animals with increased opportunity to access food in later feed drops [14]. Commercial gestating sows are commonly fed a restricted diet that is unlikely to result in satiation [24], particularly after only one quarter of the daily ration has been delivered. A more likely explanation to the findings of Rault et al. [14] is that feeding motivation declines over subsequent feed drops resulting in reduced appetitive behavior (e.g., those associated with the localisation of food) being displayed after all the food has been consumed. Indeed, the present study indicates that sows consume the majority of feed within 5–10 min of delivery when floor fed four times per day, but Rault et al. [14] observed feeding behavior for 30 min post-feeding. By contrast, the results of the present study, and others [23], show that although aggression declines over subsequent feed drops, dominant sows nonetheless monopolise the main feeding area after each feed drop, for at least as long as feed remains available.

Pigs are a gregarious species, and when housed in groups establish roughly linear, and mostly stable, dominance hierarchies [25]. Submissive sows actively avoid interactions with those more dominant, and through this avoidance, the dominance hierarchy regulates priority access to resources, including food [26]. Even when fed ad libitum, high ranking sows occupy the feeding area more frequently and more often feed alone, whereas low ranking sows wait until other sows leave the feeding area before beginning their meal, and then often feed alongside others and until they are displaced [6]. In addition to restrictions on the quantity of food provided to gestating sows, floor feeding systems place considerable spatio-temporal restrictions on the availability of feed. Consequently, the space that is available to pigs to feed, and regulate social interactions when feeding, is limited. This forces low-ranking sows to either risk receiving aggression by feeding in close proximity to high ranking sows, or avoid the area where feed is available.

It is likely that the quantitative dietary restrictions imposed on gestating sows means that all sows, independently of aggression classification/hierarchy position, experience feelings of hunger and are highly motivated to feed. Hunger is primarily aroused by internal cues, but fear is primarily aroused by external stimuli [27]. When sows have to compete for access to feed, the motivation to minimise harm by avoiding aggression (i.e., fear) is in direct conflict with the motivation to approach the feeding area [28]. In such cases, the length of time an animal spends feeding is not a simple reflection of feeding tendency or motivation to feed, but also an indication of the strength of competing motivations [28]. Webster [29] described hunger as one of the two (the second being thirst) most basic, primitive and unremitting of all motivations. Thus, the level of fear within the cohort of sows that avoid the feeding area, and the consequences of that fear on animal welfare, needs to be recognised. Verdon et al. [1] found that, once a dominance hierarchy has been established, subdominant sows were more likely to experience greater stress early after mixing and submissive sows sustained the most injuries, while both categories of sow gained less weight than their dominant pen mates. The present research, along with that of Verdon et al. [1], highlights ethical questions relating to the cost (in terms of fear, stress and injuries) that floor fed sows are required to risk to access the feeding area and to feed.

Few scientific studies have assessed management alternatives for floor feeding systems over recent years. Consequently, the development of floor feeding systems have focused on infrastructure (e.g., feeder capacity) or personnel (e.g., availability) capabilities, rather than addressing animal consequences. If floor feeding systems are to be used for gestating sows, there is a requirement for further research on the management and design of these systems to ensure that (1) all sows can access the feeding area and (2) the welfare of sows while feeding is safeguarded. One strategy to promote satiety and reduce competition in floor fed sows fed multiple times per day could be to reduce the time between feed drops. Schneider et al. [16] found reduced injuries and removal for injury when group-housed sows were floor fed six times (3 feed drops at 30 min intervals from 0700 and again from 1530 h) rather than twice (0700 and 1530 h) per day, although there were no effects on sow weight or variation in sow weight. Another strategy is to extend the length of meals [30,31]. Lawrence and Terlouw [32] proposed that feed restriction and the inability to express foraging behavior are a major cause of the development of oral stereotypies in sows. High-fiber diets, similar in dietary energy and major nutrient levels to a standard control diet, fed to sows markedly increases feeding time [24], and Robert et al. [33] showed that this increased feeding time accounted for much of the differences in level of stereotypies between diets.

## 5. Conclusions

The hypothesis that feeding sows their daily ration over multiple drops per day will result in more equitable feeding opportunities in later drops is rejected. Dominant sows spent the most time feeding in the location of the pen where the majority of feed was distributed. Subordinate (i.e., any sow that is not dominant) sows fed opportunistically, consuming what they can from between and around dominant females. However, submissive sows spent more time than subdominant and dominant sows avoiding the feeding area. These relationships were true regardless of day, feed drop within days, and gestation. Further research on the management and design of floor feeding systems is necessary to ensure that all group-housed sows are able to access the feeding area and feed with less risk to their welfare, or, alternatively, determine whether variation in feed intake is a feature of floor feeding systems per se. Since competition between group-housed sows exists for all commercial production systems, in terms of accessing the feeding area or maintaining position in the feeding area, this necessary research has broad implications for most feeding systems.

## Figures and Tables

**Table 1:** Number of pens recorded per feed drop (FD) and day, and total sows recorded per day, in gestations 1 and 2.

Recording Day	Number of Pens Recorded				Number of Sows Recorded ^1^
	FD 1	FD 2	FD 3	FD 4	
Gestation 1					
Day 2	20	20	20	15	200
Day 9	20	20	20	20	197
Day 51	15	15	15	10	140
Gestation 2					
Day 2	20	20	20	15	199
Day 9	20	20	20	20	196
Day 51	20	20	19	14	177

^1^ The number of sows recorded during at least one feed drop.
